# Fully Bioabsorbable Capacitor as an Energy Storage Unit for Implantable Medical Electronics

**DOI:** 10.1002/advs.201801625

**Published:** 2019-01-22

**Authors:** Hu Li, Chaochao Zhao, Xinxin Wang, Jianping Meng, Yang Zou, Sehrish Noreen, Luming Zhao, Zhuo Liu, Han Ouyang, Puchuan Tan, Min Yu, Yubo Fan, Zhong Lin Wang, Zhou Li

**Affiliations:** ^1^ CAS Center for Excellence in Nanoscience Beijing Key Laboratory of Micro‐nano Energy and Sensor Beijing Institute of Nanoenergy and Nanosystems Chinese Academy of Sciences Beijing 100083 P. R. China; ^2^ Beijing Advanced Innovation Centre for Biomedical Engineering Beihang University Key Laboratory for Biomechanics and Mechanobiology of Ministry of Education School of Biological Science and Medical Engineering Beihang University Beijing 100083 P. R. China; ^3^ National Research Center for Rehabilitation Technical Aids Beijing 100176 P. R. China; ^4^ School of Nanoscience and Technology University of Chinese Academy of Sciences Beijing 100049 P. R. China; ^5^ School of Materials Science and Engineering Georgia Institute of Technology Atlanta GA 30332‐0245 USA; ^6^ Center on Nanoenergy Research School of Physical Science and Technology Guangxi University Nanning 530004 P. R. China

**Keywords:** biodegradable, bioresorbable, capacitor, energy storage, implantable medical device

## Abstract

Implantable medical electronic devices are usually powered by batteries or capacitors, which have to be removed from the body after completing their function due to their non‐biodegradable property. Here, a fully bioabsorbable capacitor (BC) is developed for life‐time implantation. The BC has a symmetrical layer‐by‐layer structure, including polylactic acid (PLA) supporting substrate, PLA nanopillar arrays, self‐assembled zinc oxide nanoporous layer, and polyvinyl alcohol/phosphate buffer solution (PVA/PBS) hydrogel. The as‐fabricated BC can not only work normally in air but also in a liquid environment, including PBS and the animal body. Long‐term normal work time is achieved to 30 days in PBS and 50 days in Sprague–Dawley (SD) rats. The work time of BC in the liquid environment is tunable from days to weeks by adopting different encapsulations along BC edges. Capacitance retention of 70% is achieved after 3000 cycles. Three BCs in series can light up 15 green light‐emitting diodes (LEDs) in vivo. Additionally, after completing its mission, the BC can be fully degraded in vivo and reabsorbed by a SD rat. Considering its performance, the developed BC has a great potential as a fully bioabsorbable power source for transient electronics and implantable medical devices.

Implantable medical devices (IMDs) provide an effective therapeutic method for ever‐increasing neurological and cardiovascular diseases.^1^ These IMDs mainly involve biosensors, pacemakers, defibrillators, and stimulators used for deep brain, bone, or nerve.^2^ Long‐term in vivo diagnosis and therapy have very high demand on the high reliability, good biocompatibility, and miniaturization of the IMDs. The implanted power sources of existing IMDs mainly depend on non‐biodegradable batteries.^3^ These batteries often cause internal heat, capacity loss, and battery failure in long‐term service. Once these implanted batteries complete their functions, the patients have to undergo another surgery to take out them and as a result bear huge economic burdens as well as suffer pains. These restricted conditions above necessitate new innovations on power sources of IMDs.^4^

Transient electronics often consisted of biodegradable metals and organic polymers, which often serve as passive implants and drug‐delivery vehicles in biomedical researches. These transient electronic devices can be fully or partly dissolved in body fluid or phosphate buffer solution (PBS) in a controlled fashion.^5^ In 2014, Yin et al. have reported the first water‐activated biodegradable primary batteries using bioresorbable metal foils. The single cell has a low output voltage (0.4 V), and the integrated cell array has a large dimension (3 cm × 2 cm × 1.3 cm).[[qv: 5b]] In 2015, Fu et al. developed a rechargeable and flexible device with output voltage of 2.8 V. The component materials (electrode, current collector, and substrate) of the device have a very fast dissolution rate within a few seconds or minutes.[[qv: 5c]] In 2017, Lee et al. studied the capacitive behaviors of tungsten (W), iron (Fe), and molybdenum (Mo) during repetitive cycling. A biodegradable Mo‐based device was fabricated with a fast dissolution rate (less than 3 days).[[qv: 5d]] These factors limited their long‐term application in the liquid environment in vitro and implantable medical field in vivo. Long‐term normal work was vital for practical liquid operations in vitro and implantation in vivo. In 2016, Zheng et al. have reported a fully biodegradable triboelectric nanogenerator (TENG) for in vivo applications,^6^ whereas TENGs generate an alternating current (AC),^7^ which should be rectified and stored in non‐degradable commercial capacitors for the next step of usage.

Herein, we developed a fully bioabsorbable capacitor (BC) as a feasible energy storage unit for transient electronics in liquid environments in vitro and implantable medical devices in vivo. Biodegradable iron (Fe) film was used as current collector of BC.^8^ The BC has a layer‐by‐layer structure. Nanopillars were fabricated on the surface of polylactic acid (PLA) supporting substrate to act as adhesion promotor for Fe film.^9^ PLA with nanopillars (NP‐PLA) provided strong adhesion force to anchor the current collector. Self‐assembled nanoporous zinc oxide (ZnO) layer was used for ions storage by an evaporation‐driven self‐assembly technology.^10^ Hydrogel of PBS/PVA (polyvinyl alcohol) acted as the electrolyte and separator in charging/discharging process.^11^ The as‐fabricated BC can not only work in air but also in a liquid environment, including phosphate buffered saline (PBS) in vitro and animal body in vivo. Additionally, the BC achieved a high voltage of 1.5 V and a long work time of 30 days in PBS and 50 days in Sprague–Dawley (SD) rats. The work time of BC was tunable from days to weeks by using different edge encapsulation strategies in liquid environment. Three BCs in series can light up 15 green light‐emitting diodes (LEDs). After the BC completed its mission, it can be fully degraded and resorbed by SD rats. Considering its biocompatibility, biodegradability, bioabsorbability, long‐term normal function, and tunable work time in the liquid environment in vitro and in vivo, this developed BC has a great application potential as a power source for future transient electronics and implantable medical devices.

As shown in **Figure** **1**a, the developed BC has a multilayered structure, including NP‐PLA film, current collector (Fe), ZnO layer, and PVA/PBS hydrogel. The NP‐PLA film protected the whole device from external environment, which in turn ensured the stable electrochemical performance in vitro and in vivo. The nanopillars on NP‐PLA surface was carefully prepared by using titanium foil and hydrothermal post‐treatment in PBS at 80 °C (Figure 1b). The titanium foil provided a template for the formation of an uneven surface. The hydrothermal reaction caused chain scission of PLA and made the macromolecular chain break down into small molecules and then dissolved in PBS.^12^ The hydrothermal reaction promoted the growth of nanopillars on PLA surface. The transparent PLA film became opaque NP‐PLA film due to transition from amorphous phase to crystalline phase (Figure 1c).^13^ The nanopillars tightly anchored the deposited Fe layer and provided a strong adhesion force during magnetron sputtering (Figure 1b,dVI; Figure S1, Supporting Information), which avoided the commonly used toxic adhesion promoter of chromium. Nanoporous ZnO layer was prepared for energy storage at room temperature using an evaporation‐driven self‐assembly technology (Figure 1b).[[qv: 10c]] Then, hydrogel of PVA/PBS was dropwise added on the ZnO layer serving as solid‐state electrolyte and separator (Figure 1c; Figure S2, Supporting Information). The inner space of the as‐fabricated BC has a sandwich structure (Figure 1d,i). The materials in upper and lower layers were self‐assembled ZnO. The middle layer was PVA/PBS hydrogel (EDX mapping, Figure 1dii). The evaporation‐driven self‐assembly process induced ZnO particles to self‐assemble into a nanoporous layer (Figure 1dIV). The thickness of self‐assembled ZnO layer was about 18 µm (Figure 1diii). This porous structure offered a large, solvated ion accessible surface area for charge storage (Figure 1dV). The deposited Fe film has a thickness of about 200 nm, which was anchored tightly on the PLA nanopillars (Figure 1dVI).

**Figure 1:**
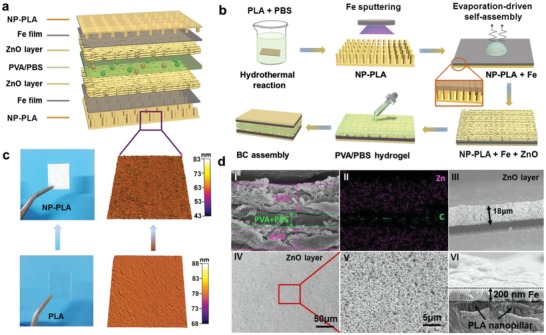
Structure, morphology, and preparation of BC. a) Structure schematic of the as‐fabricated BC. The red and green balls in PVA/PBS hydrogel represent cations and anions, respectively. b) Schematic diagram of the preparation process of BC using evaporation‐driven self‐assembly technology on NP‐PLA supporting substrate. c) Pictures and AFM topographies of PLA and NP‐PLA films. d) EDX mapping and SEM images of self‐assembled ZnO layer, PVA/PBS hydrogel, Fe film, and PLA nanopillars. The purple and green dots in EDX mapping represent zinc element and carbon element originated from ZnO layer and PVA/PBS hydrogel, respectively.

To investigate the basic capacitive performance of BC in air, cyclic voltammetry (CV) measurement was carried out at different scan rates from 10 to 300 mV s^−1^ (**Figure** **2**a). The rectangular shape of CV curves indicated good pesudocapacitive performance of ZnO.^14^ The galvanostatic charge/discharge (GCD) curves showed a symmetric triangular shape from 0.01 to 1 mA cm^−2^.^14^ It has demonstrated a pesudocapacitive behavior of faradaic charge storage (Figure 2b), which was in good agreement with the CV results. To explore the safe operation voltage of BC, the voltages of CV curves were scanned from 1 to 2 V. As shown in Figure 2c, when the scan voltages were below 1.5 V, the CV curves were close to rectangular shape, and no significant current increase appeared until 1.8 V. The rapid increase in current implied the decomposition of water molecule or chlorine evolution.^15^ Therefore, it is reasonable to think that the safe operation voltage of BC can reach up to 1.5 V in practical application.

**Figure 2:**
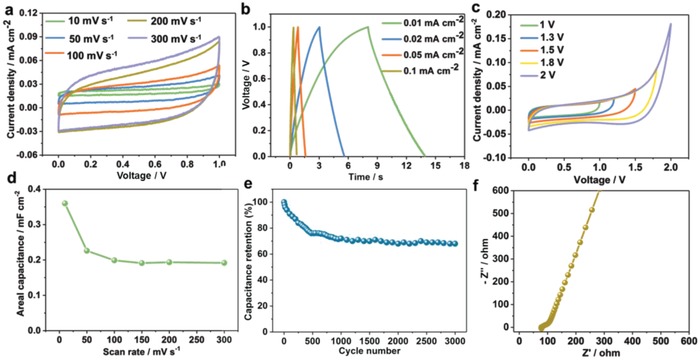
Basic capacitive performance of BC in air condition. a) CV curves at different scan rates. b) GCD curves at different current densities. c) Test of safe operation voltage window. d) Areal capacitance variation with different scan rates. e) Cycle stability test with the current density of 0.02 mA cm^−2^ at 1 V. f) Nyquist plot reflecting the impedance characteristics of BC.

The areal capacitance (Figure 2d) of BC was recalculated to mF cm^−2^ according to the following formula:^15^

(1) $$C_{a}=\frac{\int IdV}{v\cdot \Delta V\cdot A}$$

where *C*_a_ (mF cm^−2^) is the areal capacitance, *I* (A) is the response current, *v* (V s^−1^) is the voltage scan rate, Δ*V* (V) is the voltage window, and *A* (cm^−2^) is the effective area of active material (ZnO) layer. The areal capacitance values first decreased and then remained stable with the increasement of scan rate (Figure 2d). This feature can be attributed to the higher ionic migration delay in charging and discharging at higher scan rates.^15^, ^16^ The areal capacitance was also calculated using charge/discharge curves, and the capacitance showed a similar variation trend with the CV curves (Figure S3, Supporting Information). Cyclic test showed that the capacitance of BC first decreased in early 700 cycles and then remained stable for further 3000 cycles (Figure 2e). The decrease in capacitance could be attributed to the insufficient activation of active materials. A capacitance retention of about 70% was achieved after 3000 cycles, indicating good cyclic performance of the fabricated BC. The capacitance was calculated using formula (2). In the repetitive charge/discharge, the Coulombic efficiency slightly increased with cycle numbers (Figure S4, Supporting Information), which can be attributed to the effective activation and pseudocapacitance of ZnO and Fe electrode. These data indicated that weak reversible surface redox occurred between metal‐oxide and the hydrogel electrolyte during the repetitive cycling.^17^

The impedance characteristic of BC was measured using electrochemical impedance spectroscopy (EIS) equipment in a frequency range from 10 mHz to 100 kHz. In the complex plane, the real component (Z′) and imaginary component (−Z″) showed the ohmic property and capacitive property, respectively. The theoretical Nyquist plot of a capacitor contains three parts that are relevant to the frequencies in theory. In higher frequency region, the capacitor behaves like a pure resistor. In the low‐frequency region, the Nyquist plot sharply increases until it resembles a vertical line parallel to the −Z″ part, indicating the typical capacitive performance. In the medium‐frequency region, it can reflect the influence of electrode porosity on capacitive property. When it decreases from higher frequency, the signals penetrate into the porous active materials deeper and deeper, then more and more surface areas become available for ion adsorption. The medium‐frequency region is in connection with the electrolyte penetration in the porous structure. This region is commonly called the Warburg curve. The inclination angle of EIS curve partly reflects the structural integrity of the electrode.^15^, ^18^

In the low‐frequency region, the EIS curve showed an approximate vertical linear trend, which indicates a good capacitive behavior (Figure 2f). The intercept of the real axis (Z′) reveals the equivalent series resistance (ESR) of capacitor corresponding to the resistance of active materials and the electrolyte, the contact resistance between them, the interfacial resistance at the active materials and the current collector, and the diffusion resistance of the ions in PVA/PBS hydrogel. The ESR of the as‐fabricated BC was about 76 Ω from intercept of Z′. According to previous reports,^6^, ^19^ the AC energy of TENG should be stored in commercial capacitor and converted into direct current for the next step of usage.^20^ Here, we demonstrated the feasibility of storing the AC energy of TENG in our fabricated BC. The detailed results are shown in Figures S5–S8 in the Supporting Information.

For implantable medical devices, it is very important to keep its normal work in a liquid environment. To prove the feasibility of BC for in vivo implantation, we conducted the short‐term and long‐term capacitive performance test in PBS at the physiological temperature of 37 °C. The edges of BC devices for short‐term (**Figure** **3**a–d) and long‐term (Figure 3e–h) tests were encapsulated with PVA and PLA polymer solutions, respectively. The areal capacitance was calculated using the following formula[[qv: 15b]]:

(2) $$C_{a}=\frac{\int_{t0}^{t}i\;dt}{V\times A}$$

where *t*_o_ and *t* are the starting time and end time of discharge, respectively. *i* is the discharge current. V is the voltage range of capacitor, *A* is the effective area of the active material (ZnO) layer.

**Figure 3:**
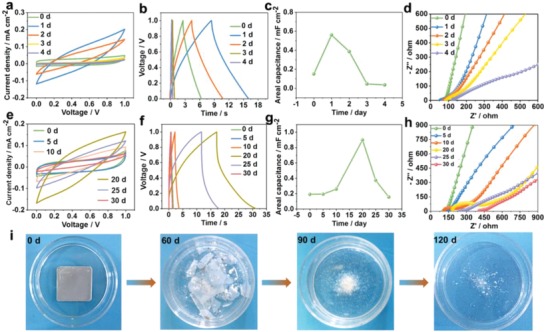
Capacitive performance and biodegradability of BC in the liquid environment in vitro. Electrochemical performance test of BC for a–d) a short‐term work and e–h) a long‐term work in PBS at 37 °C. i) In vitro degradation of BC in PBS in a cell‐culture dish (inner diameter: 35 mm) at 37 °C. The current densities in (b) and (f) were 0.02 and 0.1 mA cm^−2^, respectively.

For the short‐term test, the CV curves at 0 day showed a rectangular shape. It indicated a very good capacitive behavior of the as‐fabricated BC in the initial stage (Figure 3a). Subsequently, the shape of CV curves turned from a rectangular shape to an inclined shuttle shape at 0 and 1 day, then the inclination angle of CV curves decreased gradually to zero from 1 to 4 days. Meanwhile, the time of charging/discharging increased from 6.3 to 16 s at 0 and 1 day, then decreased from 16 to 0.5 s at 1 and 4 days (Figure 3b). Variations in both trends of CV and GCD curves were in agreement with those of areal capacitance. As shown in Figure 3c, the calculated capacitance first increased from 0 day (0.15 mF cm^−2^) to 1 day (0.56 mF cm^−2^), then decreased from 1 to 4 days (0.04 mF cm^−2^). The capacitance increment reached to the maximum value of 3.7‐fold at 1 day (Figure S9, Supporting Information). The increased capacitance can be attributed to the PBS infiltration and corrosion of Fe electrode.[[qv: 5d,18b]] Infiltration of small amounts of PBS can reduce the ionic migration resistance in PVA hydrogel to some extent.^15^ The corrosion of Fe electrode partially contributed to faradaic pseudocapacitance.[[qv: 5d,21]] After water deeply infiltrated into PVA/PBS hydrogel, the electrode and structure integrity were influenced (Figure S10, Supporting Information). The areal capacitance decreased sharply from 1 to 4 days due to the structural failure. The hypothesis was verified by the ESR and inclination angle from EIS curves (Figure 3d). The freshly fabricated BC was placed for a certain time to remove the excess water (see details in Experimental Section, Supporting Information). At this stage, the BC has a larger ESR of about 77 Ω at 0 day. With continuous infiltration of water into PVA/PBS hydrogel (Figure S10, Supporting Information), the state of solid‐state hydrogel turned into semi‐solid state at 1 day. Some PVA/PBS hydrogel dissolved into water. The dissolution path showed a radial diffusion mode (1 day in Figure S10, Supporting Information). The charge migration in PVA/PBS hydrogel became easier with a smaller ESR of 55 Ω (1 day). In the following immersion, the hydrogel dissolved into PBS gradually, the ZnO layer fell off from the electrode, small parts of Fe electrode were broken, the integrity of BC structure came apart, the above combined action made ESR of BC increased to about 122 Ω at 4 days subsequently. The inclination angles of EIS curves decreased gradually from a vertical angle at 0 day to about 25° at 4 days due to destruction of the electrode.^18^

To achieve a longer work time of BC in a liquid environment, PLA polymer solution was used as another edge encapsulation material to protect BC from external environment. The overall variation trend of capacitive behaviors was similar to that of PVA‐encapsulated BC, whereas the work time was obviously improved to 30 days (Figure 3e–h). Additionally, the initial capacitive performance kept stable at the first 5 days from the CV curves, GCD curves, and capacitance values at 0 and 5 days. This feature can be ascribed to the high reliability and robustness of PLA polymer chain. In the later immersion, the rectangular shape of CV curves gradually turned into inclined shuttle‐like shape (Figure 3e). The symmetric GCD curves turned into asymmetric shape, and a rapid voltage drop formed along the discharging curves at 20th and 25th days (Figure 3f; Figure S11, Supporting Information). This phenomenon might arise from the increase of distance between electrodes, partial corrosion of electrode, and weak contact between active material and electrode due to long‐term immersion in PBS. The combined actions resulted in the increase of overall resistance of BC, which in turn caused a significant voltage drop in discharging process. The increased integral resistances are shown in Figure 3h. From the intercepts of the real axis (Z′), the ESR gradually increased from about 85 to 390 Ω at 0 and 30 days (Figure 3h). At the 20th day, the areal capacitance reached to the maximum value (0.9 mF cm^−2^ in Figure 3g). And the maximum capacitance increment of 4.7‐fold was also obtained at the 20th day (Figure S12, Supporting Information). At this point, the pseudocapacitance from the corrosion of electrode dominated the capacitive performance, which could be verified by the EIS plots. The EIS curve at the 20th day has an obvious semicircle in the high‐frequency region (Figure 3h), indicating the process of electrode reaction was dominated by kinetic control of charge transfer.^18^ After 20 days, the destruction of the whole BC device dominated the capacitive performance. The shape of CV curves gradually recovered to the rectangular shape with a very small area, the GCD curves recovered to symmetric shape with a very short charging/discharging time, and the areal capacitance sharply decreased to near zero. These results can be ascribed to the combined actions of further corrosion and detachment of electrode, hydrolysis of PLA supporting substrate, and the solvation of hydrogel electrolyte.

As shown in Figure 3i, the biodegradability and real‐time change of BC were demonstrated by immersing BC in PBS at 37 °C for 4 months. The whole BC device kept structural integrity in the initial stage. After 2 months, the PLA supporting substrates broke down into fragments due to the hydrolysis reaction.^22^ Fe electrode was almost totally corroded in PBS. After immersion for a longer time, rapid autocatalytic hydrolysis and bulk degradation happened, PLA film became into powder, and the hydrogel and ZnO disappeared in constantly renewing PBS (3 months). After 4 months, almost all the BC device degraded in PBS, indicating good in vitro biodegradability of BC in a normal or physiological environment. Additionally, the mass loss of BC with a fast degradation rate at 80 °C was also simulated to study the material degradation behavior (Figure S13, Supporting Information). The mass loss at the early stage can be attributed to the fast dissolution of water‐soluble PVA/PBS hydrogel. In the following time, the mass loss can be ascribed to the decomposition of PLA, Fe, and ZnO, and the repetitive replacement of PBS solvent. The BC was completely degraded after 15 days.

Biocompatibility of implantable device is an essential requirement in practical implantation process, which largely relies on the encapsulation strategy and constituent materials. Here, L929 cells were used to evaluate the biocompatibility of Fe film, PLA film, ZnO powder, and BC device at the cellular level. For Fe film and PLA film, the L929 cells were cultured on their surfaces. The cell compatibility tests of the whole BC device and constituent ZnO powder were carried out using their extraction solution, respectively.^23^ As shown in **Figure** **4**, immunofluorescence staining was carried out to investigate the attachment, proliferation, and morphology of the cultured L929 cells at different times. After cultured for 1 day, L929 cells showed a good attachment and cellular morphology as single cell for all groups. In subsequent incubation, the cellular density of L929 cells obviously increased with incubation time. All the L929 cells showed a good filamentous and stretched morphology (2 days). Some cell clusters formed and even grew into a confluent cell monolayer (3 days). All the results coincided with those of relative viabilities of L929 cells by 3‐(4,5‐dimethylthiazol‐2‐yl)‐2,5‐diphenyltetrazolium bromide (MTT) assay test (Figure S14, Supporting Information). The cultured L929 cells showed a high viability (>95%) from 1 day to 3 days, which showed a good biocompatibility of the constituent materials of BC.

**Figure 4:**
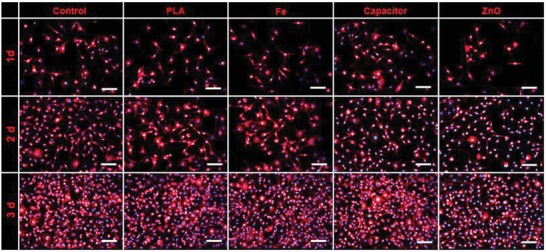
Biocompatibility of constituent materials of BC. Attachment, proliferation, and morphology of the L929 cells at different time. Scale bars: 100 µm.

To evaluate the in vivo performance and bioabsorbability, PLA‐encapsulated BCs were implanted in the dorsal subcutaneous region of SD rats (**Figure** **5**a,b). All the implantation operations were performed strictly in accordance to the “Beijing Administration Rule of Laboratory Animals” and the national standard “Laboratory Animal Requirements of Environment and Housing Facilities (GB 14925‐2001).” All the BCs were sterilized by γ irradiation (^60^Co/25 kGy) before implantation. The implanted BC showed good capacitive performance in vivo up to 50 days (Figure 5c,d; Figure S15, Supporting Information). The variation trend of electrochemical curves was similar to that of PLA‐encapsulated BC in PBS. The increment of capacitance reached up to 7.5‐fold of the original capacitance value (Figure S16, Supporting Information), whereas the work time of BC in vivo (50 days) was longer than that in PBS (30 days). The reason was probably that the amount of body fluid of SD rats was much smaller than the used PBS in vitro, which slowed the hydrolysis rate of PLA supporting substrate. Additionally, the fibrous capsule around BC may also contribute to the structural integrity, which in turn extended the work time of BC in vivo. The areal energy density (*E*_a_) and power density (*P*_a_) were calculated by the following formulas[[qv: 15b]]:

(3) $$E_{a}=\frac{C_{a\;}\times \;V^{2}}{2\;\times \;3600}$$

(4) $$P_{a}=\frac{E_{a}\;\times \;3600}{t_{\mathrm{discharge}}}$$

where *V* and *t*_discharge_ are the operation voltage and discharge time of BC, respectively. The *E*_a_ of 0.153 µW h cm^−2^ was obtained at a power density of 27 µW cm^−2^, and the *P*_a_ of 0.526 mW cm^−2^ was obtained at an energy density of 0.013 µW h cm^−2^ (Figure S17, Supporting Information). By comparison, the obtained capacitive performance of BC was comparable to those of the other reported supercapacitors (Table S1, Supporting Information). And the capacitive performance of BC was superior in a liquid environment and suitable for implantable medical electronics.

**Figure 5:**
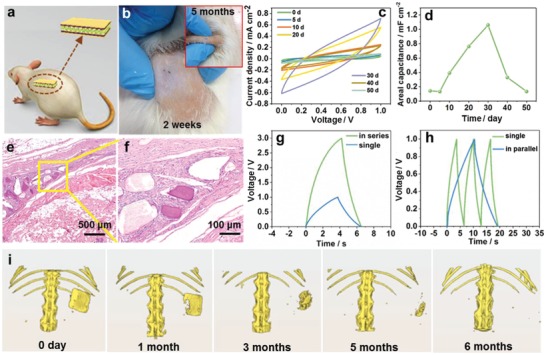
Bioabsorbability evaluation and capacitive performance of BC in vivo. a) Implantation diagram of a BC in the dorsal subcutaneous region of a SD rat. b) Pictures of implanted site containing a BC before and after degradation at different time. c) CV curves of an implanted BC for 50 days. d) Variations of areal capacitance of the implanted BC for 50 days. e,f) Hematoxylin and eosin (H&E) staining of excised tissue from implanted site after 6 months. g,h) GCD curves of BCs in series and in parallel, respectively. i) In vivo biodegradation of BC in a SD rat for 6 months via micro‐CT imaging system. The images were reconstructed from micro‐CT system and processed with pseudocolor technology. The dimension of implanted BC was 1.5 cm × 1.5 cm × 1 mm.

As shown in Figure 5b, the wound healed well without obvious inflammation after 2 weeks. After implanted for 5 months, the implanted BC disappeared from view (inset in Figure 5b). The real‐time shape changes of BC in the SD rat was monitored by micro‐computed tomography (micro‐CT) system (Figure 5i). In the degradation process, ZnO gradually leaked out from the edges of broken BC due to hydrolysis of PLA and resorbed by the SD rat through metabolism in the following months. After the BC disappeared from view using micro‐CT (6 months), the SD rat was euthanized. The subcutaneous tissue containing PLA residuals at the implantation site was taken for further histological study (Figure 5e,f). The Hematoxylin and Eosin (H&E) stained histologic section showed that little degraded PLA residuals existed between subcutaneous layer and muscle layer (Figure 5e). The normal regenerated tissue has grown and filled the gaps between the residues. Some macrophages existed around the residues to metabolize the hydrolyzed polymer (Figure 5f). No neutrophil granulocytes and obvious inflammations were observed, revealing good in vivo biocompatibility and bioabsorbability of the developed BC.

To demonstrate the practical application of BC, three BCs were assembled in series (Figure 5g) and in parallel (Figure 5h) and charged at 0.02 mA cm^−2^. Compared with a single BC with an operation voltage of 1 V, the three BCs connected in series exhibited a charge/discharge voltage of 3 V with similar charge/discharge time. The charge/discharge time of the parallel connected BCs was about three times that of a single BC at the same current density. These results indicated a good maintenance in capacitive performance of the developed BC. Then, three BCs in series were implanted in the dorsal subcutaneous region of a SD rat and charged to 3.2 V (Figure S18, Supporting Information) to power 15 green LEDs. The LED pattern was immediately lighted up successfully (Figure S19, Supporting Information). These results indicated that the developed BC in this work has great potential as a fully bioabsorbable power source for transient electronics and implantable medical devices.

In summary, we successfully developed a fully biodegradable and BC as an energy storage unit. All the constituent materials of BC showed good biocompatibility and bioabsorbability. The fabricated nanopillars on NP‐PLA surface provided strong adhesion for current collector. BC showed good capacitive performance in liquid environments and achieved long‐term normal work of 30 days in PBS in vitro and 50 days in SD rats in vivo. The work time of BC in liquid environment was tunable from days to weeks by adopting different edge encapsulations using PVA and PLA polymer solution. A high operation voltage of 1.5 V was achieved. A capacitance retention of about 70% was obtained for 3000 cycles. After implanted tandem BCs in SD rat, it can light up 15 green LEDs as a power source immediately. After completing its mission, the developed BC can be fully degraded and resorbed by the SD rat. The developed BC provided a feasible energy solution to work as a fully bioabsorbable power source for future in vivo implanted medical devices. After the power source finished its mission, it can be fully resorbed by the body, and the patients can be exempted from the second surgery and financial burden. In other liquid environments, the BC has an application potential for environmentally friendly electronics for underwater operations that cannot be realized by conventional techniques.

## Conflict of Interest

The authors declare no conflict of interest.

## Supporting information

Supplementary

## References

[1] a) I. M. Mosa , A. Pattammattel , K. Kadimisetty , P. Pande , M. F. El‐Kady , G. W. Bishop , M. Novak , R. B. Kaner , A. K. Basu , C. V. Kumar , J. F. Rusling , Adv. Energy Mater. 2017, 7, 1700358; 29104523 10.1002/aenm.201700358 PMC5667682

[2] a) S. J. A. Majerus , S. L. Garverick , M. A. Suster , P. C. Fletter , M. S. Damaser , ACM J. Emerging Technol. Comput. Syst. 2012, 8, 1; 10.1145/2180878.2180883 PMC4712728 26778926

[3] a) M. Irimia‐Vladu , Chem. Soc. Rev. 2014, 43, 588; 24121237 10.1039/c3cs60235d

[4] a) S. W. Hwang , H. Tao , D. H. Kim , H. Y. Cheng , J. K. Song , E. Rill , M. A. Brenckle , B. Panilaitis , S. M. Won , Y. S. Kim , Y. M. Song , K. J. Yu , A. Ameen , R. Li , Y. W. Su , M. M. Yang , D. L. Kaplan , M. R. Zakin , M. J. Slepian , Y. G. Huang , F. G. Omenetto , J. A. Rogers , Science 2012, 337, 1640; 23019646 10.1126/science.1226325 PMC3786576

[5] a) S.‐W. Hwang , H. Tao , D.‐H. Kim , H. Y. Cheng , J.‐K. Song , E. Rill , M. A. Brenckle , B. Panilaitis , S. M. Won , Y.‐S. Kim , Y. M. Song , K. J. Yu , A. Ameen , R. Li , Y. W. Su , M. M. Yang , D. L. Kaplan , M. R. Zakin , M. J. Slepian , Y. G. Huang , F. G. Omenetto , J. A. Rogers , Science 2012, 337, 1640; 23019646 10.1126/science.1226325 PMC3786576

[6] Q. Zheng , Y. Zou , Y. L. Zhang , Z. Liu , B. J. Shi , X. X. Wang , Y. M. Jin , H. Ouyang , Z. Li , Z. L. Wang , Sci. Adv. 2016, 2, e1501478. 26973876 10.1126/sciadv.1501478 PMC4783121

[7] a) W. Jiang , H. Li , Z. Liu , Z. Li , J. J. Tian , B. J. Shi , Y. Zou , H. Ouyang , C. C. Zhao , L. M. Zhao , R. Sun , H. R. Zheng , Y. B. Fan , Z. L. Wang , Z. Li , Adv. Mater. 2018, 30, 1801895; 10.1002/adma.201801895 29947102

[8] a) M. Peustera , C. Hesse , T. Schloo , C. Fink , P. Beerbaum , C. V. Schnakenburg , Biomaterials 2006, 27, 4955; 16765434 10.1016/j.biomaterials.2006.05.029

[9] a) D. D. Silva , M. Kaduri , M. Poley , O. Adir , N. Krinsky , J. Shainsky‐Roitman , A. Schroeder , Chem. Eng. J. 2018, 340, 9; 10.1016/j.cej.2018.01.010 PMC6682490 31384170

[10] a) M. Grzelczak , J. Vermant , E. M. Furst , L. M. Liz‐Marzan , ACS Nano 2010, 4, 3591; 20568710 10.1021/nn100869j

[11] M. Chaouat , C. L. Visage , W. E. Baille , B. Escoubet , F. Chaubet , M. A. Mateescu ,, D. Letourneur , Adv. Funct. Mater. 2008, 18, 2855.

[12] a) B. S. Ndazi , S. Karlsson , eXPRESS Polym. Lett. 2011, 5, 119;

[13] a) T. Tábi , I. E. Sajó , F. Szabó , A. S. Luyt , J. G. Kovács , eXPRESS Polym. Lett. 2010, 4, 659;

[14] a) C. H. Kim , B.‐H. Kim , J. Power Sources 2015, 274, 512;

[15] a) H. Li , X. X. Wang , W. Jiang , H. Y. Fu , X. Q. Liang , K. Zhang , Z. Li , C. C. Zhao , H. Q. Feng , J. Nie , R. P. Liu , G. Zhou , Y. B. Fan , Z. Li , Adv. Mater. Interfaces 2018, 5, 1701648;

[16] K. Fic , G. Lota , M. Meller , E. Frackowiak , Energy Environ. Sci. 2012, 5, 5842. 10.1002/cssc.201200227 22692854

[17] W. Li , S. Wang , L. Xin , M. Wu , X. Lou , J. Mater. Chem. A 2016, 4, 7700.

[18] a) S. M. Park , J. S. Yoo , Anal. Chem. 2003, 75, 455A; 14619851

[19] J. Wang , X. H. Li , Y. L. Zi , S. H. Wang , Z. L. Li , L. Zheng , F. Yi , S. M. Li , Z. L. Wang , Adv. Mater. 2015, 27, 4830. 26175123 10.1002/adma.201501934

[20] a) H. Y. Guo , M.‐H. Yeh , Y.‐C. Lai , Y. L. Zi , C. S. Wu , Z. Wen , C. G. Hu , Z. L. Wang , ACS Nano 2016, 10, 10580; 27934070 10.1021/acsnano.6b06621

[21] S. F. Zhu , N. Huang , L. Xu , Y. Zhang , H. Q. Liu , H. Sun , Y. X. Leng , Mater. Sci. Eng., C 2009, 29, 1589.

[22] a) G. H. Yew , A. M. Mohd Yusof , Z. A. Mohd Ishak , U. S. Ishiaku , Polym. Degrad. Stab. 2005, 90, 488;

[23] a) D. H. Yan , G. F. Yin , Z. B. Huang , L. Li , X. M. Liao , X. C. Chen , Y. D. Yao , B. Q. Hao , Langmuir 2011, 27, 13206; 21932858 10.1021/la2008107

