# Supplementary material for: Fully Bioabsorbable Capacitor as an Energy Storage Unit for Implantable Medical Electronics
Source: Adv Sci (Weinh). 2019 Jan 22;6(6):1801625. doi: 10.1002/advs.201801625 (PMC6425441; doi:10.1002/advs.201801625)
Supplement: Supplementary file 1 — Supplementary [file ADVS-6-1801625-s001.pdf]

Copyright WILEY-VCH Verlag GmbH & Co. KGaA, 69469 Weinheim, Germany, 2019.

ADVANCED SCIENCE
Open Access

Supporting Information

for *Adv. Sci.,* DOI: 10.1002/advs.201801625

Fully Bioabsorbable Capacitor as an Energy Storage Unit for Implantable Medical Electronics

Hu Li, Chaochao Zhao, Xinxin Wang, Jianping Meng, Yang Zou, Sehrish Noreen, Luming Zhao, Zhuo Liu, Han Ouyang, Puchuan Tan, Min Yu, Yubo Fan, Zhong Lin Wang,* and Zhou Li**

Supporting Information for

Fully bioabsorbable capacitor as an energy storage unit for implantable medical electronics

Hu Li, Chaochao Zhao, Xinxin Wang, Jianping Meng, Yang Zou, Sehrish Noreen, Luming Zhao, Zhuo Liu, Han Ouyang, Puchuan Tan, Min Yu, Yubo Fan, * *Zhong Lin Wang,* * *and Zhou Li* *

H. Li, C. C. Zhao, Dr. X. X. Wang, Dr. J. P. Meng, Y. Zou, S. Noreen, L. M. Zhao, Z. Liu, H. Ouyang, P. C. Tan, Dr. M. Yu, Prof. Z. L. Wang, Prof. Z. Li

CAS Center for Excellence in Nanoscience, Beijing Key Laboratory of Micro-nano Energy and Sensor, Beijing Institute of Nanoenergy and Nanosystems, Chinese Academy of Sciences, Beijing, 100083, P. R. China.

School of Nanoscience and Technology, University of Chinese Academy of Sciences, Beijing 100049, P. R. China.

Email: zli@binn.cas.cn

H. Li, Z. Liu, Prof. Y. B. Fan

Beijing Advanced Innovation Centre for Biomedical Engineering, Beihang University, Key Laboratory for Biomechanics and Mechanobiology of Ministry of Education, School of Biological Science and Medical Engineering, Beihang University, Beijing 100083, P. R. China.

National Research Center for Rehabilitation Technical Aids, Beijing, 100176, P. R. China;

Email: yubofan@buaa.edu.cn

Prof. Z. Li

Center on Nanoenergy Research, School of Physical Science and Technology, Guangxi University, Nanning, 530004, China

Prof. Z. L. Wang

School of Materials Science and Engineering, Georgia Institute of Technology, Atlanta, Georgia 30332-0245, United States.

Email: zhong.wang@mse.gatech.edu

Table S1. Comparison of capacitive performance with other reported supercapacitors.

Electrode material	Electrolyte	Operation voltage	Workable in water?	Workable in vivo?	Degradable?	C_a (mF cm^{-2})	E_a (μWh cm^{-2})	P_a (mW cm^{-2})	Ref.
CNF/RGO /CNTs	PVA/H_2SO_4	1 V	--	--	--	216	28.4	9.5	[1]
Graphene /PEDOT:PSS	PVA/H_3PO_4	0.8 V	--	--	--	212.9	6.8	0.2	[2]
RGO fiber	PVA/H_3PO_4	0.8 V	--	--	--	186.8	4.8	0.2	[2]
MnO_2@Si NWs	Li-ion doped ionic liquid	2.2 V	--	--	--	13	9.1	0.388	[3]
Au/MnO_2/Au	PVA/$LiClO_4$	0.8 V	--	--	--	11.9	1.1	0.1	[4]
Onion-like Carbon	Et_4NBF_4-PC	3 V	--	--	--	1.7	0.28	11	[5]
Mo/MoO_3	NaCl/Agarose	0.8 V	--	--	yes	1.6	0.14	1	[6]
Fe/ZnO/gel/ ZnO/Fe	PVA/PBS	1.5 V	yes 30 days	yes 50 days	yes	1.1	0.153	0.526	Our work

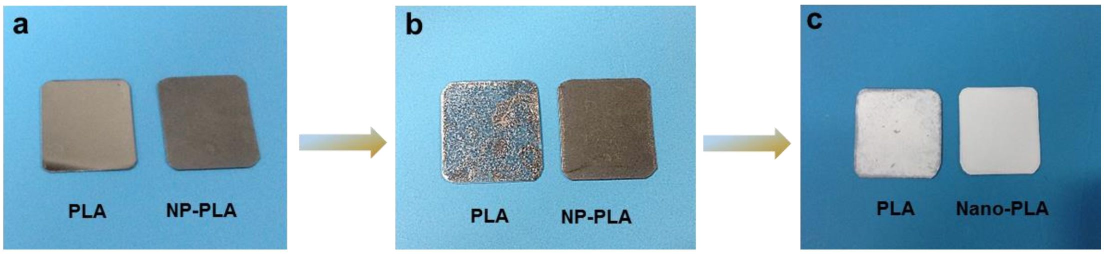

Figure S1. Comparison of adhesion force of Fe electrodes on PLA and NP-PLA films. (a) Pictures of freshly fabricated PLA and NP-PLA films. (b) Pictures of PLA and NP-PLA after addition of ethyl alcohol on their surfaces. (c) Pictures of PLA and NP-PLA after self-assembly of ZnO layer using supersaturated dispersion solution of ZnO and ethyl alcohol.

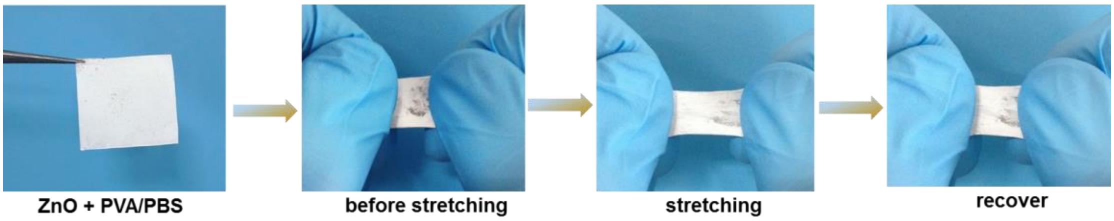

Figure S2. PVA/PBS hydrogel peeled from Fe electrode. Some of ZnO layer covered on the surface of PVA/PBS hydrogel. The prepared hydrogel was stretchable and recoverable to some extent.

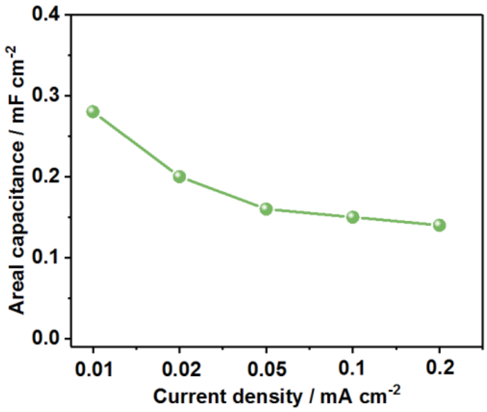

Figure S3. Areal capacitance variation with different current density in air.

The areal capacitance in Figure S3 was calculated using the following formula:

$$C_a = \frac{\int_{t0}^{t} i\,dt}{V \times A}$$

where t_o and t is the starting time and end time of discharge, respectively. i is the discharge current. V is the voltage range of the capacitor, A is the effective area of the active material (ZnO) layer.

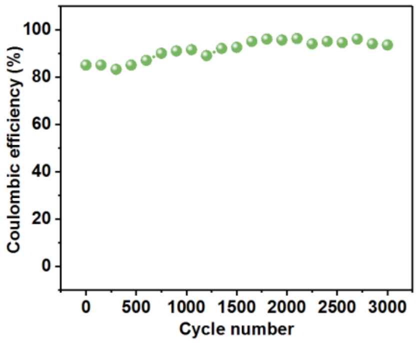

Figure S4. Coulombic efficiency variation of the as-fabricated BC during repetitive charge/discharge.

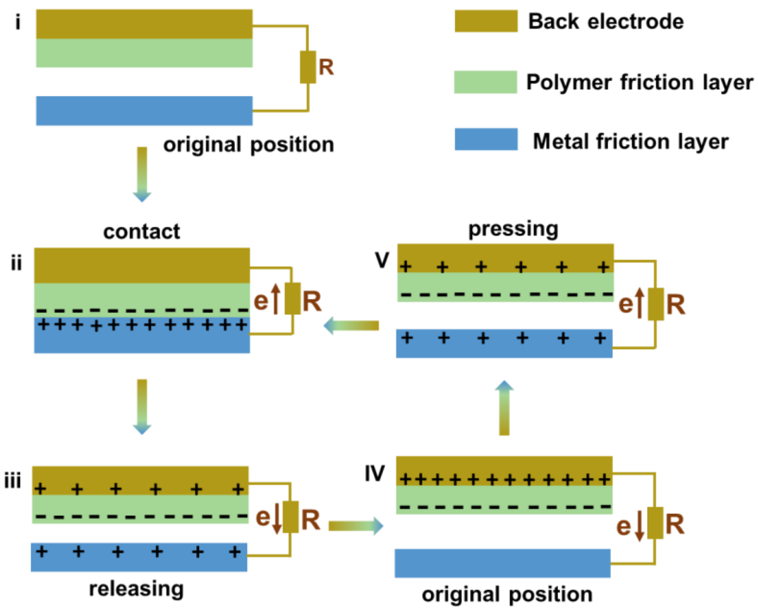

Figure S5. Working principle of TENG with the contact-separation mode.

Figure S5 showed the working principle of triboelectric nanogenerator (TENG). When two fraction layers contact with each other, equal numbers of charges of different polarity formed on the contact surface (ii). After releasing the TENG, a potential drop between back electrode and metal friction layer impelled the free electrons transferred from back electrode to metal friction layer to balance the electrostatic field (iii and IV). When the external force made the friction layers contact again, the induced electrons flowed back and created a reverse current (V and ii). The periodic contact and separation of polymer and metal friction layers generated an AC output signal in the external circuit.

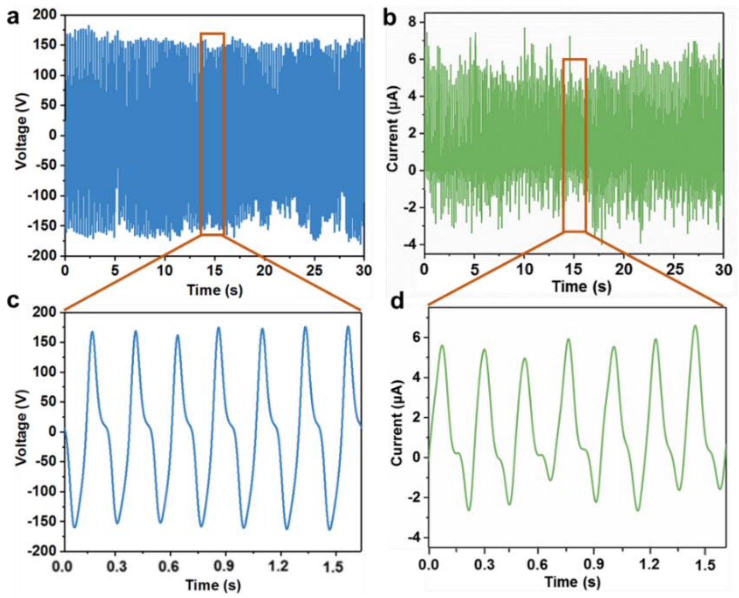

Figure S6. Output voltage (a, c) and current (b, d) of TENG before rectification at a frequency of 4.24 Hz.

As shown in **Figure S6**, the forward and reverse voltages of TENG were about 175 V and -150 V, respectively. The forward and reverse currents of TENG were about 6 μA and -2.5 μA, respectively.

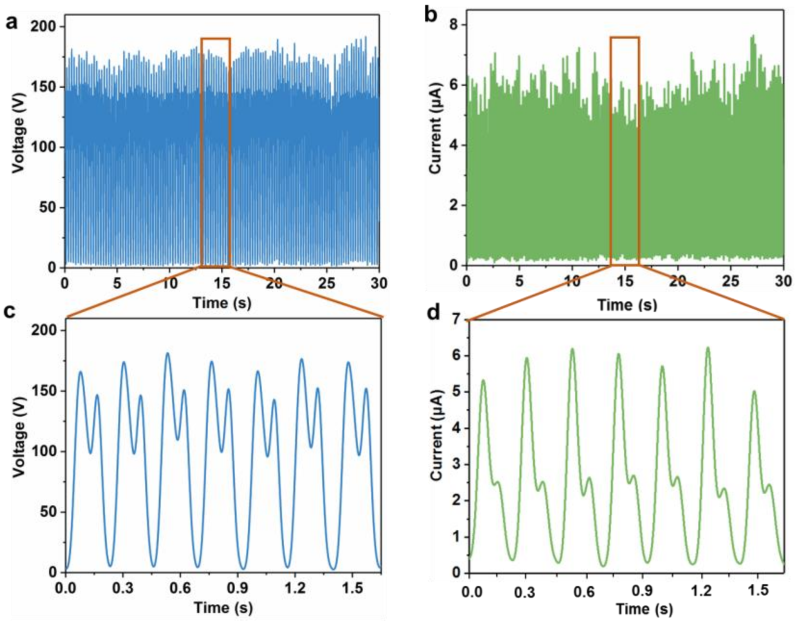

Figure S7. Output voltage (a, c) and current (b, d) of TENG after rectification at a frequency of 4.24 Hz.

After rectification, the electrical output signals have a constant direction (**Figure S7**). The values of electrical output remain the same with primary state.

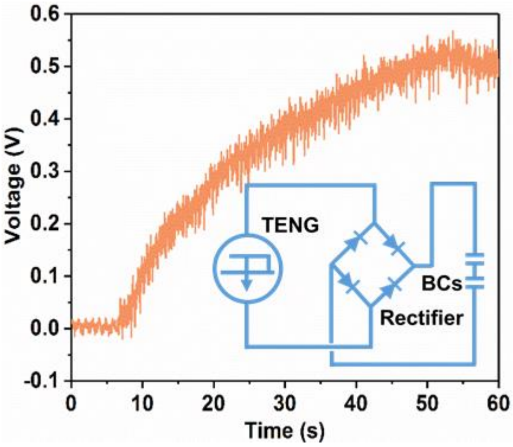

Figure S8. Charging curve of two tandem BCs using TENG. The inset represents corresponding circuit diagram of charging BCs.

After connecting the rectified TENG with two tandem BCs, the BCs can be charged to 0.55 V in 50s (**Figure S8**). It indicated that the as-fabricated BC can effectively store rectified AC energy from TENG for the next step of usage.

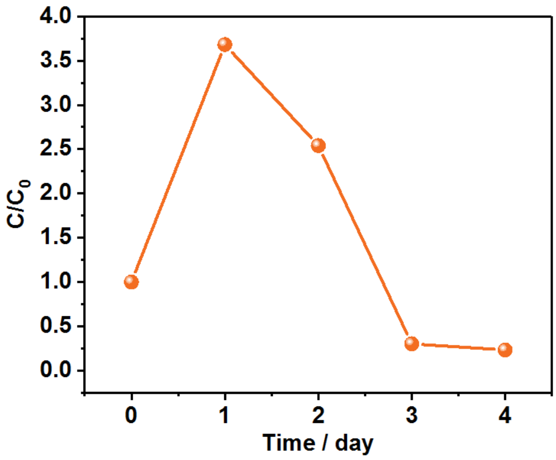

Figure S9. Increment of capacitance of PVA-encapsulated BC in PBS at 37℃.

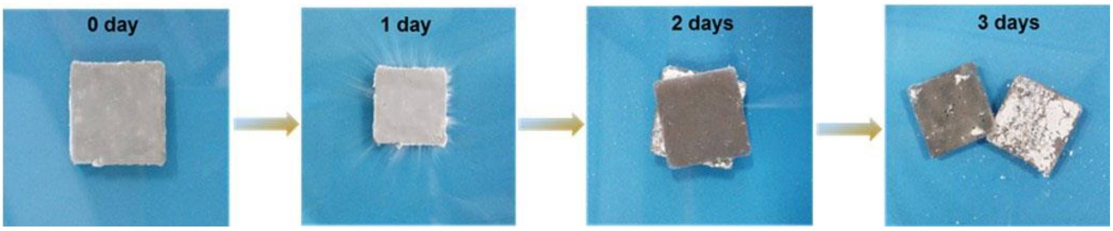

Figure S10. Demonstration of BC encapsulated with PVA in PBS solution for a short-term operation at 37 ℃. At the 1st day, the ZnO layer diffused in PBS along with PVA hydrogel. At the 2nd day, the integrity of BC device was broken. At the 3rd day, few Fe electrode fell off from PLA supporting substrate around the edges and centers.

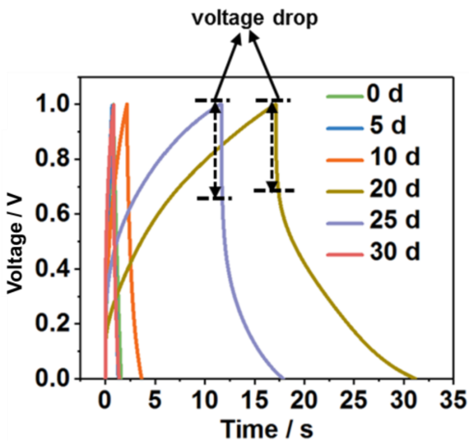

Figure S11. Voltage drop of PLA encapsulated BC in PBS at 20 d and 25 d.

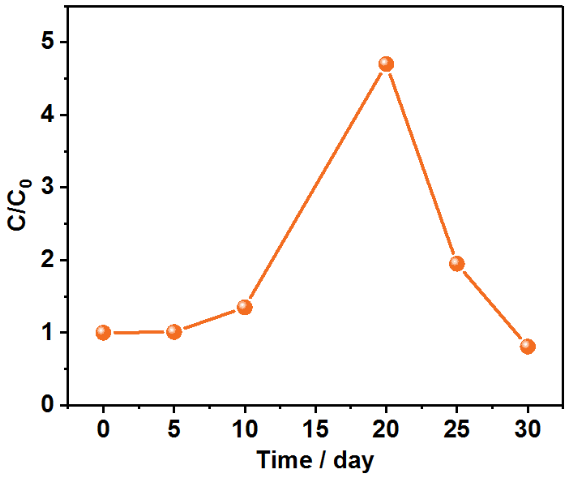

Figure S12. Increment of capacitance of PLA-encapsulated BC in PBS at 37℃.

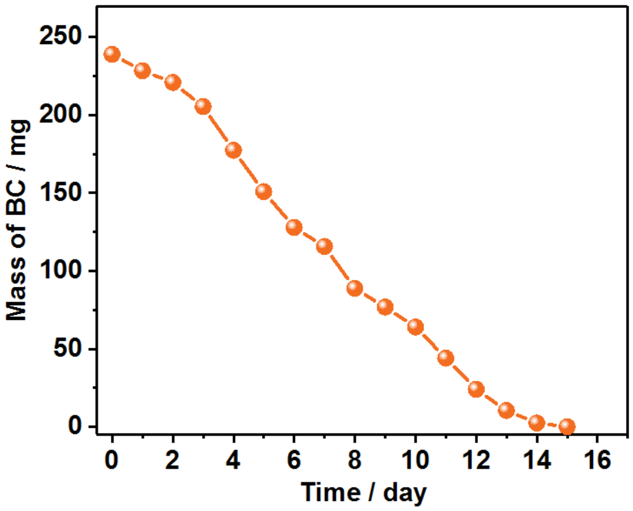

Figure S13. Mass loss of BC in PBS at 80 ℃.

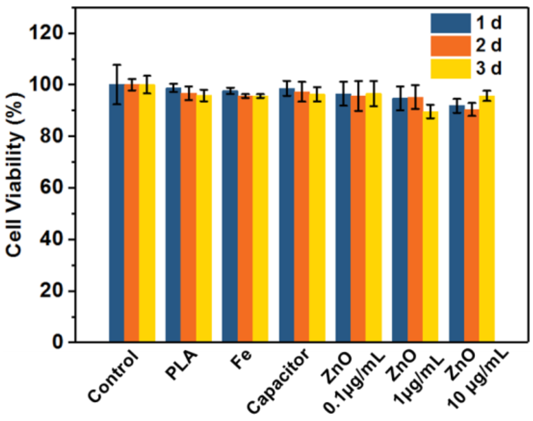

Figure S14. MTT analysis test of the cultured L929 cells. The error bars represent standard deviation.

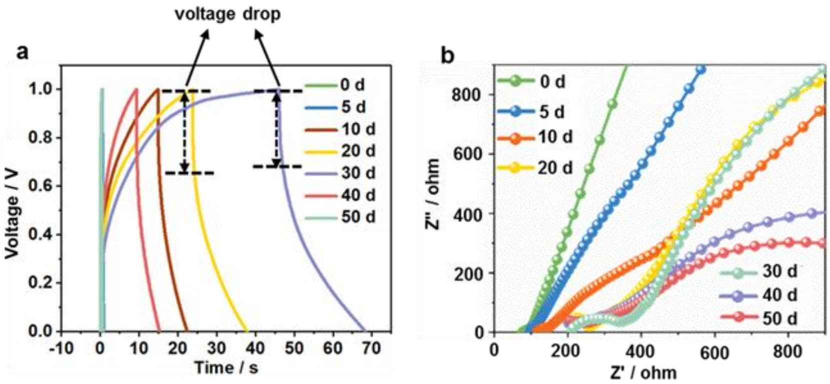

Figure S15. Electrochemical performance of PLA encapsulated BC in the SD rat for 60 days. (a) GCD curves of BC in the SD rat for 50 days. (b) EIS plots of BC in SD rats for 50 days.

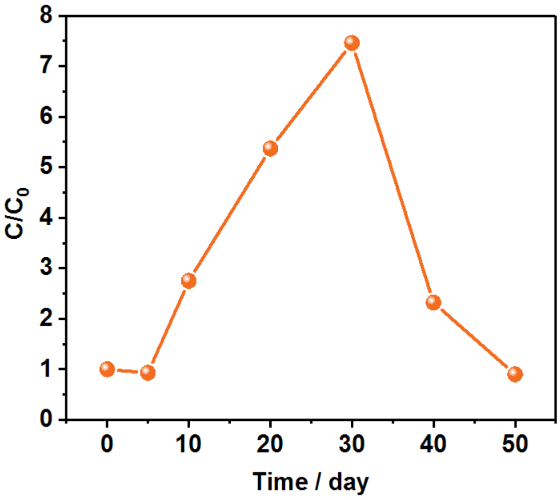

Figure S16. Increment of capacitance of PLA-encapsulated BC in the SD rat at 37℃.

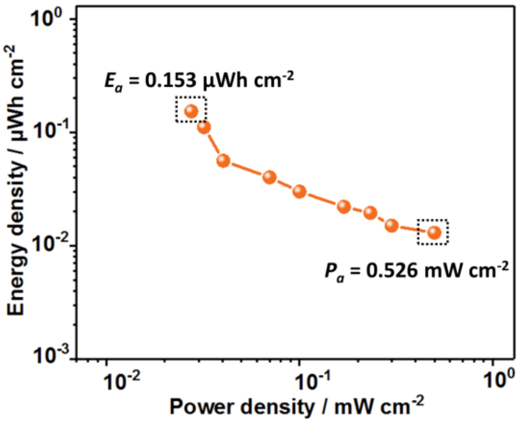

Figure S17. Ragone plot of power and energy density of BC.

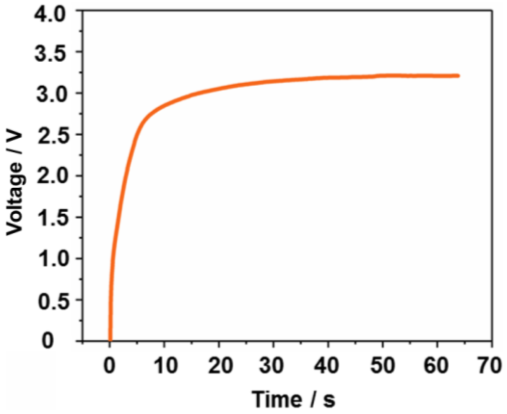

Figure S18. Charging curve of three BCs in series for lighting up the green LED pattern.

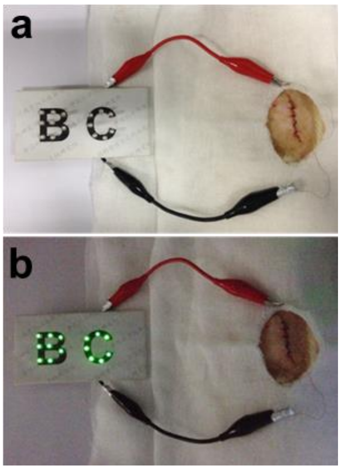

Figure S19. Practical demonstration of lighting up a green LED pattern using BCs in series in a SD rat. LED pattern (a) before and (b) after powered by BCs.

Experimental Section

Preparation of NP-PLA film and Fe electrode: PLA granules (Sigma-Aldrich) were placed on a titanium foil heated at 250 ℃ using a magnetic stirring apparatus (MS7-H550-Pro, Dragon Lab). When the PLA granules melt into a transparent liquid, another titanium foil was pressed on the molten polymer liquid. After the molten polymer liquid was compressed into a thin film with an expected thickness (about 0.4 mm), the titanium foils were taken down off the magnetic stirring apparatus and immersed into deionized water (25 ℃) until fully cool off. Then the PLA film was peeled off from the cooled titanium foil and tailored to a proper size (1.5 cm × 1.5 cm). Finally, the PLA films were put into PBS (1 X) at 80 ℃ in a drying oven for two days. Particularly, to accelerate the *in vivo* degradation of the capacitor, the PLA

films for implantation experiment were treated in PBS solution (1 X) at 80 ℃ for 2.5 days. The hydrothermal reaction made nanopillars grow on the surfaces of PLA film.

Fe film (thickness, 200 nm) was deposited on the surface of Nano-PLA by vacuum magnetron sputtering (PVD75 Proline, Kurt J. Lesker® Company) at 100W for 30 minutes. The nanopillars were embedded in the deposited Fe film. The deposited Fe film, in turn, was tightly anchored on the surface of NP-PLA. The nanostructure avoided the commonly used harmful adhesion promoter of Cr layer.

Preparation of PVA hydrogel: The PBS (concentration, 10 X) was purchased from gibco® by life technologies for cell culture. Then the PBS was diluted 10 times with deionized water to achieve a proper concentration (1 X). The PVA powder (2 g) was dissolved in the as-prepared PBS (1 X, 20 ml) by sonication at 80℃ until it formed a homogeneous sticky solution. Then the solution was cooled down to room temperature and became a clear and transparent hydrogel.

Self-assembly of ZnO layer and assembly of BC: All the ZnO powders (Sigma-Alorich) used in this experiment were washed with plenty of deionized water and ethyl alcohol (75 vol%) successively by ultrasonication. The suspension solution was centrifuged and transferred to a sterilized vial for the standby application. To achieve a fast self-assembly of ZnO layer on Fe film, the pure ethyl alcohol was applied as dispersing agent. Fifteen milligrams of ZnO powder were added into the pure ethyl alcohol solution (1 ml) and then treated with sonication at 100 W for 20 minutes at room temperature.

Before the self-assembly of ZnO layer, two leads were connected respectively to the electrodes. The lead end was covered with a thin PLA film (thickness, about 60 μm) to protect electrodes from connection during the assembly of BC. Then the as-prepared ZnO suspension solution (volume, 1 ml) was dropwise added onto the surface of Fe film at room temperature. When ethyl alcohol solution completely evaporated into the air, ZnO powder was self-assembled into a nanostructured layer driven by evaporation. Then one drop of the as-prepared PVA hydrogel (volume, 0.05 ml) was added onto the surface of ZnO layer and flattened with a silicon wafer to make the hydrogel fully cover the ZnO layer. Then kept the sample in a clean bench

until the PVA hydrogel completely dried out and formed a thin film. The dried PVA hydrogel film fixed the ZnO powder on Fe electrode and kept its structure intact in the following assembly process. Subsequently, another two drops of PVA hydrogel (volume, 0.1 ml) was secondly added onto the dried PVA hydrogel film. Two of the as-prepared samples were assembled immediately as a sandwich structure and clamped with an alligator clip for about one hour until the extruded PVA hydrogel around the capacitor edges completely dried out.

Encapsulation of BC edges: PVA hydrogel and PLA polymer solution were used as the encapsulation agent, respectively. The PVA hydrogel was prepared by above-mentioned method. The PLA polymer solution was prepared by blending of PLA granules and trichloromethane. The mass concentration of PLA was 5 wt%. Then the edges of BC for *in vitro* test were encapsulated with PVA hydrogel and PLA polymer solution using a glass dropper. The edges of BC for *in vivo* test were encapsulated with PLA polymer solution. After the PVA hydrogel and PLA polymer solution completely dried out, the as-prepared capacitors can be used for further experiment.

Fabrication of TENG with vertical contact-separation mode: To fabricate the used TENG, two acrylic plates were shaped by a laser cutter to serve as substrates with dimensions of 10 cm × 10 cm × 4 mm. Four holes were drilled at four corners of acrylic plates for spring installation. A layer of 100 nm copper was deposited on Kapton film to act as back electrode. The fabricated Kapton film (dimension: 7.5 cm × 7.5 cm × 100 μm) was assembled on one acrylic substrate. An aluminum foil (dimension: 7.5 cm × 7.5 cm × 200 μm) was assembled on the other acrylic substrate. Subsequently, four springs was anchored to connect the top and bottom acrylic substrates. Finally, two lead wires were used to connect the back electrode and aluminum foil for electric measurement.

Material characterization and electrochemical measurement: The electrochemical properties of the as-fabricated BC were investigated in a two-electrode configuration using electrochemical workstation (CHI660E) and impedance/gain-phase analyzer (solartron, SI1260) at room temperature. BCs were put in PBS solution (1 X) for *in*

vitro test at room temperature. After the electrochemical measurement was completed, the capacitors were kept in the constant temperature incubator (37 ℃). The implanted capacitor was measured in the anesthetized SD rats at room temperature. The BC tested in air was also conducted at room temperature.

The surface morphologies of PLA/NP-PLA films and cross section of the as-prepared BCs were characterized using SEM (HITACHI, SU8020) and AFM (Asylum Research, MFP-3D). Before the SEM characterization, the samples were treated with gold sputtering at 20 mA for 30s with Sputter Coater (Cressington108Auto). The element analysis was conducted by energy dispersive X-ray (EDX; IXRF SYSTEMS, Model 550i) mapping at 20 kV and 10 μA.

***In vitro* biodegradation simulation:** The as-prepared BCs were placed in a petri dish with PBS solution at 37 ℃ in the constant temperature incubator. The edges of the as-prepared capacitor were encapsulated with PLA polymer solution (5 wt%). The PBS solution in the petri dish was replaced every 24 hours.

Cell culture: PLA film, BC device and Fe film were fixed on the bottom of a 24-well culture plate. The L929 cells (1×10^6 cell/well) were seeded on material surfaces and cultured in Dulbecco's modified Eagle's medium (DMEM, Gibco) containing 10% fetal bovine serum (FBS, Gibco) and 1% penicillin/streptomycin (Gibco). The L929 cells were incubated in a cell incubator with a humidity atmosphere containing 5% CO_2 at 37 ℃ for 24 h, 48 h and 72 h, respectively. For ZnO power, aqueous extractions with different concentrations (0, 0.1, 1, 10 μg mL^{-1}) were prepared for L929 cell culture. The concentration of 0 μg mL^{-1} was set as the control group.

Cellular Viability Test (MTT) assay: The cell viability and proliferation of L929 cells were evaluated by MTT assay. After co-culturing for 24 h, 48 h and 72 h, the culture mediums of L929 cells were replaced with 900 μL DMEM and 100 μL MTT solution (5mg/mL). Then incubate for 4 h in a humidified atmosphere with 5% of CO_2 at 37 ℃, the supernatant solution was replaced by using DMSO to dissolve the deposited purple formazan granules. The optical density was measured using an ELIASA at the wavelength of 490 nm. Three parallel control wells were set for each group.

Cell morphology and immunofluorescent staining: After the culture process , L929 cells were fixed with immunohistochemically fixed fluid (Beyotime) for 30 min under room temperature and washed with PBS (PH, 7.4) for three times. Then the as-prepared samples were treated with 0.1% triton X-100 for 10 min and 0.1% bovine serum albumin solution for 1 h at 37 ℃, respectively. Subsequently, Alexa Fluor 594 phalloidin (1:40 dilution) and DAPI (1:400 dilution) were added successively and incubated for 1 h and 10 min, respectively. Finally, the immunofluorescent images were obtained by using an inversion fluorescence microscope (OLYMPUS, IX71).

***In Vivo* Study:** The SD rats (weight, 300 g) were obtained from Academy of Military Medical Sciences (Beijing, China) and raised for one week to make the animal adapt to the new experimental environment prior to surgery. To measure the electrochemical performance of BC *in vivo*, we implanted the capacitor in the dorsal subdermal region of a SD rat where operation and observation are more convenient. Additionally, this implanted region can efficiently avoid the lead wires being broken by the rats' scratching. The electrochemical properties of the as-fabricated BC were investigated in a two-electrode configuration using electrochemical workstation (CHI660E) and impedance/gain-phase analyzer (solartron, SI1260) at 37 ℃. The real-time degradation state of the implanted BC was observed using micro-CT system (Perkin Elmer, USA). After the BC fully disappeared from view, the SD rat was fed for another month and then euthanized for histological evaluation.

References:

[1] Q. F. Zheng, Z. Y. Cai, Z. Q. Ma and S. Q. Gong, *ACS Appl. Mater. Inter.* **2015**, 7, 3263.

[2] G.X. Qu, J. L. Cheng, X. D. Li, D. M. Yuan, P. N. Chen, X. L. Chen and B. Wang, *Adv. Mater.* **2016**, 28, 3646.

[3] D. P. Dubal, D. Aradilla, G. Bidan, P. Gentile, T. J. S. Schubert, J. Wimberg, S. Sadki and P. G. -Romero, *Sci. Rep.* **2015**, 5, 09771.

[4] H. B. Hu, Z. B. Pei, H. J. Fan and C. H. Ye, *Small* **2016**, 12, 3059.

[5] D. Pech, M. Brunet, H. Durou, P. H. Huang, V. Mochalin, Y. Gogotsi, P. -L.

Taberna and P. Simon, *Nat. Nanotechnol.* **2010**, 5, 651.

[6] G. Lee, S. -K. Kang, S. M. Won, P. Gutruf, Y. R. Jeong, J. Koo, S. -S. Lee, J. A. Rogers and J. S. Ha, *Adv. Energy Mater.* **2017**, 7, 1700157.
